# Superresolution imaging of nanoscale chromosome contacts

**DOI:** 10.1038/srep42422

**Published:** 2017-02-10

**Authors:** Yejun Wang, Prasuna Ratna, G. V. Shivashankar

**Affiliations:** 1 Mechanobiology Institute and Department of Biological Sciences, National University of Singapore 117411, Singapore; 2 FIRC Institute for Molecular Oncology (IFOM), Milan 20139, Italy

## Abstract

Co-expression of a specific group of genes requires physical associations among these genes, which form functional chromosomal contacts. While DNA fluorescence *in situ* hybridization (FISH) pinpoints the localization of genes within the 3D nuclear architecture, direct evidence of physical chromosomal contacts is still lacking. Here, we report a method for the direct visualization of transcription-dependent chromosomal contacts formed in two distinct mechanical states of cells. We prepared open chromatin spreads from isolated nuclei, ensuring 2D rendering of chromosome organization. Superresolution imaging of these chromatin spreads resolved the nanoscale organization of genome contacts. We optimized our imaging method using chromatin spreads from serum+/− cells. We then showed direct visualization of functional gene clusters targeted by YAP (Yes-associated protein) and SRF (Serum response factor) transcription factors. In addition, we showed the association of NF-κB bound gene clusters induced by TNF-α addition. Furthermore, EpiTect ChIP qPCR results showed that these nanoscale clusters were enriched with corresponding transcription factors. Taken together, our method provides a robust platform to directly visualize and study specific genome-wide chromosomal contacts.

The three-dimensional organization of the genome has been found to be critical in regulating gene expression programs in various cell types^1^,^2^,^3^,^4^,^5^. DNA is packed by histone and non-histone proteins into higher order chromosome structures within the nucleus of living cells. The spatial packing of each chromosome territory has been shown to be non-random^6^,^7^. Gene-rich chromosomes are interiorly positioned, while gene-poor chromosomes tend to be located towards the nuclear periphery. Recent evidence from chromosome FISH experiments has revealed that the relative position of chromosomes, and their intermingling, is correlated with transcription^8^,^9^. These intermingling regions, defined as chromosomal contacts, are generally formed amongst co-expressed genes. The chromosomal contacts are associated with 5 S phosphorylated RNA polymerase II (5 S RNA pol2), a transcriptionally active form of RNA pol2. In addition, such contacts are enriched with histone modification markers for decompacted chromatin, as well as specific transcription factors^10^. In addition, high-resolution genome-wide chromosome contact maps, revealed by chromosome conformation capture assays, show that specific chromosome contacts are coupled with cellular transcriptional status^11^,^12^,^13^. Furthermore, a number of studies have shown that co-expressed genes are spatially clustered within the nucleus^14^,^15^,^16^. Disruption of physical gene contacts abrogated their co-expression, confirming the requirement of gene contacts for their co-regulation. In spite of the significant importance of physical gene contacts, direct visualization of these contacts at the nanoscale level has not been achieved. This is primarily due to the inability to visualize individual genes, or a group of genes, within a crowded cellular nucleus, as well as the lack of appropriate superresolution 3D imaging methods.

Superresolution microscopy has been widely used to obtain well-resolved chromatin structures in both eukaryotic and prokaryotic cells using various labeling strategies^17^,^18^,^19^,^20^. For example, stochastic stimulation of a subset of fluorophores is achieved through either tagging histone proteins with photoactivatable fluorescent proteins^17^, or incorporating EdU labelled with photoactivatable fluorephores using the ‘click chemistry’ approach^18^. For visualizing DNA, intercalating dye YOYO-1 has been shown to efficiently label DNA though binding/unbinding kinetics in a specific reducing-oxidizing buffer^20^. With these labelling strategies, it has been found that Drosophila metaphase chromosomes contains fine filaments of ~70 nm^17^. More recently, 3D STORM combined with oligonucleotide probes has distinguished the structures among active, inactive, and repressed chromatin^19^. In addition, telomeric chromatin structures have been visualized as T-loop structures using such methods^21^. However, the direct visualization of gene clusters has still not been achieved, primarily due to the crowded nuclear environment.

In this paper, we describe an open chromatin spread system that was developed by modifying our previously described method^22^, and implementing the optimized 2D superresolution imaging technique to directly visualize the functional genomic contacts at a nanometer scale resolution. Briefly, we removed cytoplasm from attached cells, followed by fragmenting chromatin fibers using a restriction enzyme HindIII. We then immunostained isolated nuclei with short chromatin fragments for specific transcription factors and transcription machinery. After this, we applied a mechanical force to rupture the nuclei to obtain short chromatin fragments for superresolution imaging. Co-labeling of the proteins and DNA showed the preservation of transcription machinery and factors on the open chromatin spreads. The length of the short chromatin fragments matched the theoretical length after digestion, confirming the efficiency of HindIII. Superresolution imaging resolved the short chromatin fragments as structures with more than one DNA fiber associated with 5 S RNA pol2 and specific transcription factors. In serum-starved cells, we observed few contacts, whereas the amount of contacts increased significantly upon serum stimulation, indicating the functionality of the observed chromosomal contacts.

Using this method, we directly visualized specific chromosomal contacts, in particular those targeted by transcription factors/cofactors such as YAP, SRF, and NF-κB. Moreover, for cells with various geometric confinements or cytokine treatments, we observed differential levels of NF-κB target contacts. In this case, using EpiTect ChIP qPCR we also observed a consistent trend of promoter enrichment by NF-κB. It is worth noting that, by seeding cells sparsely onto glass slides, we were able to image chromosomal contacts from one cell without mixing contacts from other cells. Hence, this method also allows us to reveal heterogeneity in the level of specific contacts between cells. In summary, we report a novel open chromatin spread system that utilizes existing superresolution microscopy methods to visualize functional chromosomal contacts directly at a nanometer resolution. Our system could also serve as a robust platform to study the molecular mechanisms of contact formation.

## Results

### BALM imaging of digested chromatin fragments

**Figure 1:**
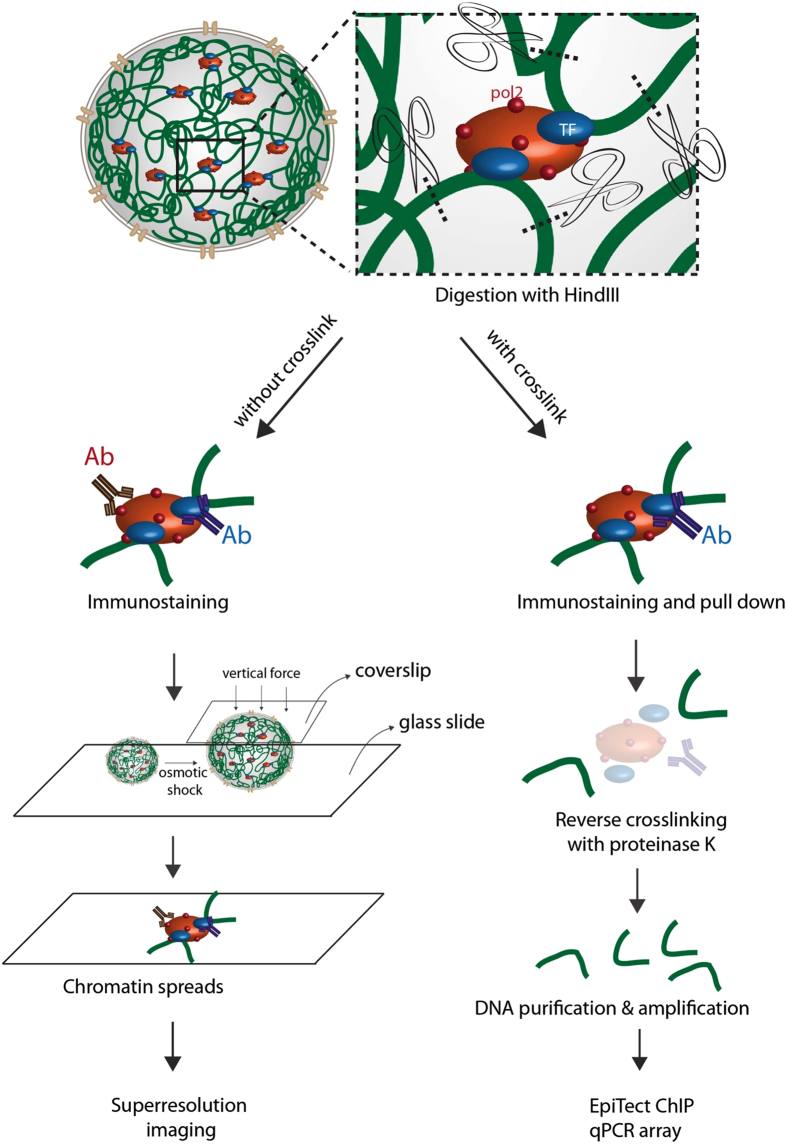
Brief overview of the chromosomal contacts imaging and EpiTect ChIP analysis. The DNA is digested by HindIII within the intact nucleus. For superresolution imaging, nuclei are not crosslinked, and the chromatin is immunostained with antibodies recognizing 5 S RNA pol2 and transcription factors (TFs). Following that, nuclei are subjected to osmotic shock, and then burst with compressive load to spread chromatin fragments on glass slides for imaging. For EpiTect ChIP analysis, crosslinked and digested chromatin fragments are pulled down with magnetic beads coated with an antibody recognizing the transcription factor NF-κB (p65). Chromosomal contacts associated with p65 are reversely crosslinked, and the DNA from the chromosomal contacts is purified and amplified, before performing EpiTect ChIP qRCR assay.

To visualize chromosomal contacts associated with specific transcription factors, we advanced our open chromatin method^22^ for chromosomal contact preparation, by incorporating one major modification. To dissociate chromosomal contacts from chromosome territories, we included a DNA digestion step using the HindIII restriction enzyme (Fig. 1), which cleaves the A-A bond within the short AAGCTT sequence. The regions protected by transcription machinery are not cleaved. To spread chromatin fragments on glass slides, we ruptured the isolated nuclei encapsulating digested chromatin fibers, by mechanically compressing the swollen nuclei (Fig. 1). Conventional microscopy imaging of digested chromatin spreads revealed short chromatin fragments with an average length of 1.5 μm. This is consistent with the theoretical length of chromatin fibers digested by HindIII (Supplementary Fig. 1), The undigested chromatin spreads, on the other hand, were long and continuous fibers (Supplementary Fig. 1a).

Unlike other biochemical techniques, such as chromosome conformation capture (3 C)-based methods^23^, we did not fix the cells in our method. This fixation step was omitted for three reasons. Firstly, the fixatives would introduce imaging artifacts of non-specific contacts. Secondly, fixed nuclei are more difficult to burst through swelling, and therefore the chromatin fragments would be poorly spread (Supplementary Fig. 2). Thirdly, the strong binding affinity between active transcription machinery and DNA preserves activated RNA pol2 and specific transcription factors on digested chromatin fragments even without fixation (Supplementary Figs 6–8).

We then visualized the digested chromatin fragments using binding activatable localization microscopy (BALM) with a resolution of ~30 nm^20^. This imaging technique was developed based on a similar principle as photoactivatable localization microscopy (PALM)^24^ and stochastic optical reconstruction microscopy (STORM)^25^. The illumination of a sparse set of fluorophores enables the detection of single molecules, and the repetitive capturing of such sparse sets of fluorophores on a total internal fluorescence (TIRF) microscopy was used to reconstruct superresolution images. Chromatin fragments were labeled with YOYO-1, a DNA intercalating dye that has an on/ off property under dynamic binding conditions in a reducing-oxidizing (ROXS) buffer: YOYO-1 that binds to DNA, in the on state, fluoresces 800–1000 times more compared to free YOYO-1 molecules^20^. Whereas conventional microscopy only resolved the chromatin fragments as blurred structures (Supplementary Fig. 3a,b), superresolution imaging of these digested fragments resolved the fine chromatin structures that contained more than one DNA fiber, which was termed as “branching DNA” based on the morphology of the structures (Supplementary Fig. 3c). To characterize these well-resolved branching DNA, we measured the thickness of the thinnest DNA fiber within one branching DNA. We found that the average thickness was 30–35 nm. Notably, we previously measured the thickness of 2-nm thick λDNA using the same imaging strategy, and found that the average thickness was also 30–35 nm^22^. Due to the localization precision limit of superresolution microscopy, the actual 30-nm fibers would appear to be fibers thicker than 30 nm. This suggests that the fibers in digested fragments are much thinner than actual 30-nm chromatin fibers^26^.

### Branching DNA was lost upon transcriptional quiescence

To check whether these branching DNA structures are formed randomly (i.e. through the overlapping of one DNA fiber on top of another), or by functional clustering, we forced cells into a transcriptionally quiescent state by serum starving them for 36 hrs. We then stimulated a proportion of the serum starved cells with 10% FBS for 12 hrs to reboot their transcription activity. Interestingly, we found that in cells cultured with a normal serum supply, ~60% of the fragments were branching DNA. However, in transcriptionally quiescent cells, only ~20% of digested chromatin fragments comprised of branching DNA (Supplementary Fig. 4). This indicated that the formation of the branching DNA visualized using superresolution microscopy is dependent on the cells transcriptional activity.

To further confirm the branching DNA structures are transcriptionally specific, we immunostained 5 S phosphorylated RNA polymerase II (5 S RNA pol2) and the serum response factor (SRF) together with branching DNA, and carried out three-color superresolution microscopy imaging. We used TetraSpeck™ beads for channel alignment and drift correction. To avoid bias in quantification, we selected a small region of interest (ROI) around the chromosomal contact in the channel of DNA, and then we checked the channels of 5 S RNA pol2 and SRF respectively to see whether there are 5 S RNA pol2 and SRF clusters in this region. ROIs with both 5 S RNA pol2 and SRF signals were scored as positive. We found that in serum stimulated cells ~30% of the chromatin fragments were branching DNA associated with 5 S RNA pol2 and SRF, whereas in serum starved cells, less than 10% were 5 S RNA pol2/SRF associated branching DNA (Supplementary Fig. 4). These results suggested that our method could resolve digested fragments as branching DNA structures, which were further proven to be functional physical chromosomal contacts. In addition, our method could detect changes in the level of SRF target chromosomal contacts following serum starvation.

### Visualization of YAP target chromosomal contacts

Next, we applied our method to cells under distinct mechanical constraints to check if this method can reveal mechanosensitive chromosomal contacts. For this, we cultured mouse fibroblasts on micro-fabricated fibronectin-coated patterns to generate different cell shapes, and examined the YAP (Yes-associated protein 1) targeted chromosomal contacts. YAP is a transcription coactivator that relays mechanical signals from ECM rigidity and cell shape to the nucleus^27^. On big anisotropic substrates, YAP predominantly localizes in nucleus and its target genes are activated, whereas on small isotropic substrates YAP is excluded from the nucleus, and resides in the cytoplasm, with its target genes being quiescent^27^.

**Figure 2:**
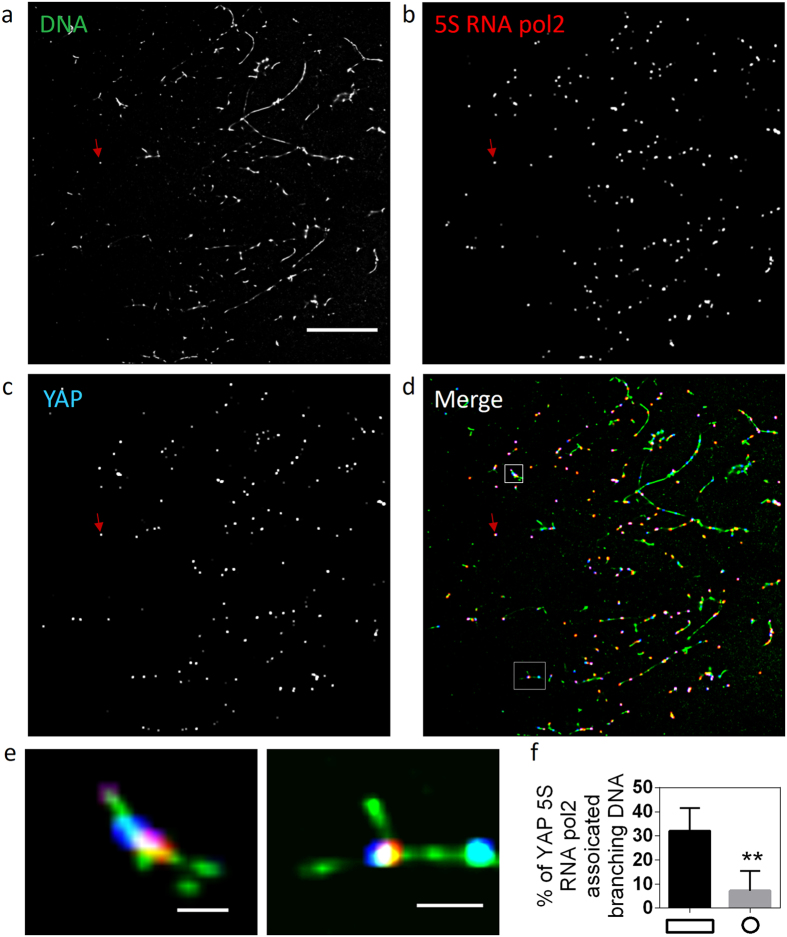
Superresolution imaging of digested chromatin fragments reveals YAP targeted chromosomal contacts. Superresolution images of (**a**) chromosomal contacts, (**b**) 5 S RNA pol2, and (**c**) YAP. The red arrows indicate a TetraSpeck^TM^ bead. Scale bar: 10 μm. (**d**) Three-color superresolution image of chromosomal contacts (green), 5 S RNA pol2 (red), and YAP (blue). The red arrow indicates a TetraSpeck^TM^ bead. (**e**) Zoomed in images of regions indicated by white boxes in (**d**). Scale bar: 200 nm. (**f**) Bar graph quantifying the percentage of chromosomal contacts associated with both 5 S RNA pol2 and YAP in cells cultured on either anisotropic (rectangle) or isotropic (circle) substrates. Data is given as mean ± SD with 10 < n < 20. **P < 0.01; Two sample student’s t test.

To visualize the YAP targeted contacts, we prepared chromatin spreads by opening up the nuclei on big anisotropic and small isotropic substrates (Supplementary Fig. 5). Conventional microscopy imaging detected more YAP associated digested fragments on big anisotropic substrates, which was consistent with the nuclear localization of YAP (Supplementary Fig. 6). Superresolution imaging of these digested fragments further revealed chromosomal contacts associated with 5 S RNA pol2 and YAP (Fig. 2a–e), with a significantly higher level of such contacts in spreading cells (Fig. 2f). As shown in Fig. 2a, some of the chromatin fragments were longer and not resolved as chromosomal contacts. These fragments were not considered in our quantification as they could be due to incomplete digestion of the DNA. These results suggested that our method could detect differential levels of YAP targeted chromosomal contacts regulated by cell shape.

### Visualization of SRF target chromosomal contacts

**Figure 3:**
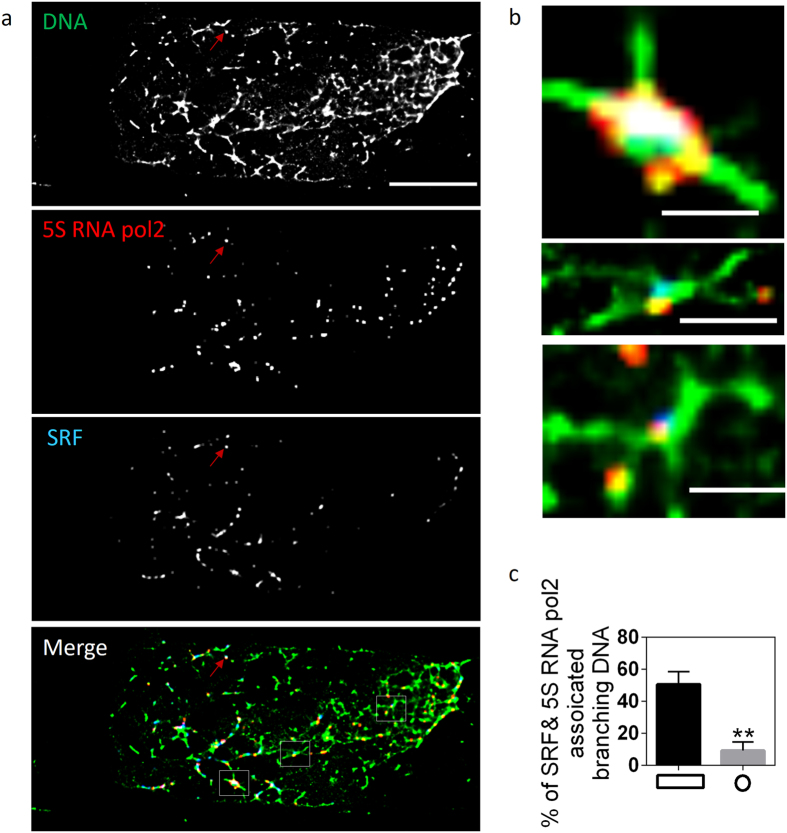
Superresolution imaging of digested chromatin fragments reveals SRF targeted chromosomal contacts. (**a**) Three-color superresolution image of chromosomal contacts (green), 5 S RNA pol2 (red), and SRF (blue). The red arrows indicate a TetraSpeck^TM^ bead. Scale bar: 10 μm. (**b**) Zoomed in images of regions outlined with white boxes in (**a**). Scale bar: 200 nm. (**c**) Bar graph quantifying the percentage of chromosomal contacts associated with both 5 S RNA pol2 and SRF in cells cultured on either anisotropic (rectangle) or isotropic (circle) substrates. Data is given as mean ± SD with 10 < n < 20. **P < 0.01; Two sample student’s t test.

To ensure that chromosome contacts, modulated by cell mechanics, were not specific to candidate transcription factors, we further tested our method by visualizing serum response factor (SRF) target chromosomal contacts. In addition to YAP transcription factors, serum response factors also localize in the nucleus to regulate their target genes, when fibroblast cells are cultured on big anisotropic substrates^28^. Consistent with this, more SRF was associated with digested chromatin fragments in spreading cells cultured on big anisotropic substrates, compared to small isotropic substrates (Fig. 3 and Supplementary Fig. 7). Superresolution imaging of these fragments revealed chromosomal contacts associated with 5 S RNA pol2 and SRF (Fig. 3a,b). Consistently, there was a significantly higher level of contacts associated with 5 S RNA pol 2 and SRF in cells cultured on anisotropic substrates (Fig. 3c) compared to isotropic cells. Moreover, since the distance between isolated nuclei was large enough to capture the digested fragments of a single nucleus (Supplementary Fig. 5), we were able to quantify the level of SRF targeted contacts within single cells. The error bars in Fig. 3c indicate the variability in the amount of SRF target contacts among different cells. However, it should be noted that the percentage of functional contacts might be under-estimated as some contacts are lost during sample preparation. These results suggest that our method can detect SRF targeted chromosomal contacts at the single-cell scale.

### Visualization of NF-κB target chromosomal contacts

Next, we checked if our method of visualizing chromosomal contact formation was sensitive to changes in the cytoplasmic to nuclear localization of transcription factors induced by cytokines. For this, we treated mechanically constrained cells with tumor necrosis factor alpha (TNF-α). TNF-α induces the nuclear localization of NF-κB transcription factors, and the subsequent expression of their target genes. As shown in supplementary Fig. 8, we first established the nuclear localization of p65, a subunit of NF-κB, in the isotropic and anisotropic cells, as well as anisotropic cells treated with TNF-α. Cells on isotropic geometries show higher nuclear localization of p65 compared to anisotropic cells. Treatment of TNF-α on anisotropic cells increases nuclear p65 levels.

**Figure 4:**
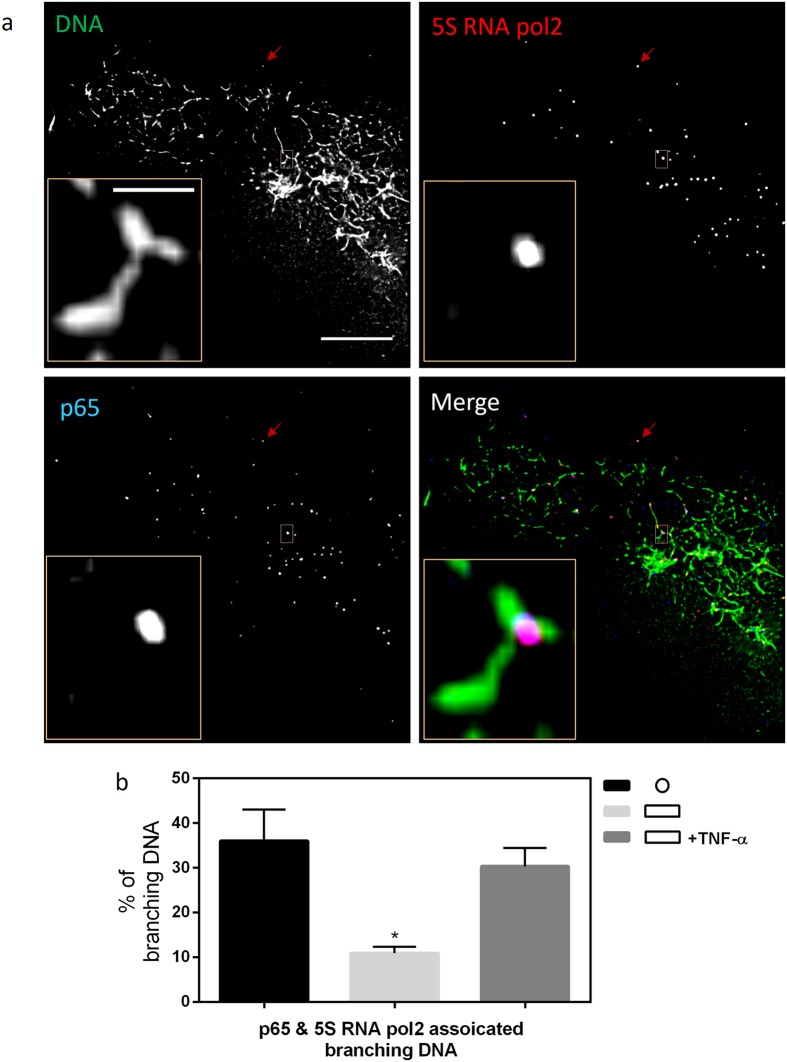
Superresolution imaging of digested chromatin fragments reveals a differential amount of p65 target chromosomal contacts in response to geometric confinement or cytokine induction. (**a**) Three-color superresolution image of chromosomal contacts (green), 5 S RNA pol2 (red), and p65 (blue). The red arrows indicate a TetraSpeck^TM^ bead. Scale bar: 10 μm. Insets: zoomed in images of the regions outlined by orange boxes. Scale bar: 200 nm. (**b**) Bar graph quantifying the percentage of chromosomal contacts associated with both 5 S RNA pol2 and p65. Data is given as mean ± SD with 10 < n < 20. *P < 0.05; one-way ANOVA test.

We then carried out superresolution imaging of chromosomal contacts in the aforementioned three cases: small isotropic cells, big anisotropic cells, and big anisotropic cells treated with TNF-α. Consistent with nuclear localization of p65, there was higher amount of digested fragments associated with p65 in isotropic cells. Addition of TNF-α to anisotropic cells also increased the amount of p65 associated chromatin fragments as visualized by conventional microscopy (Supplementary Fig. 9). Furthermore, superresolution imaging revealed the chromosomal contacts targeted by p65 (Fig. 4a). These were particularly enriched in isotropic cells, as well as anisotropic cells stimulated with TNF-α (Fig. 4b). These results suggest that with the availability of specific antibodies, our method can detect the level of chromosomal contacts targeted by various transcription factors under different conditions.

### EpiTect ChIP analysis reveals promoter occupancy of p65 on its target chromosomal contacts

To confirm the specific chromosomal contacts indeed contain particular promoter sites, we pulled down chromosomal contacts with magnetic beads coated with an antibody to p65, which served as a representative transcription factor. The DNA was then subjected to an EpiTect ChIP qPCR array with a library of primers for approximately 80 known p65 target genes (Fig. 1). This experiment reveals the promoter occupancy of p65 on a set of genes that are likely to be contained in the pulled-down chromosomal contacts.

**Figure 5:**
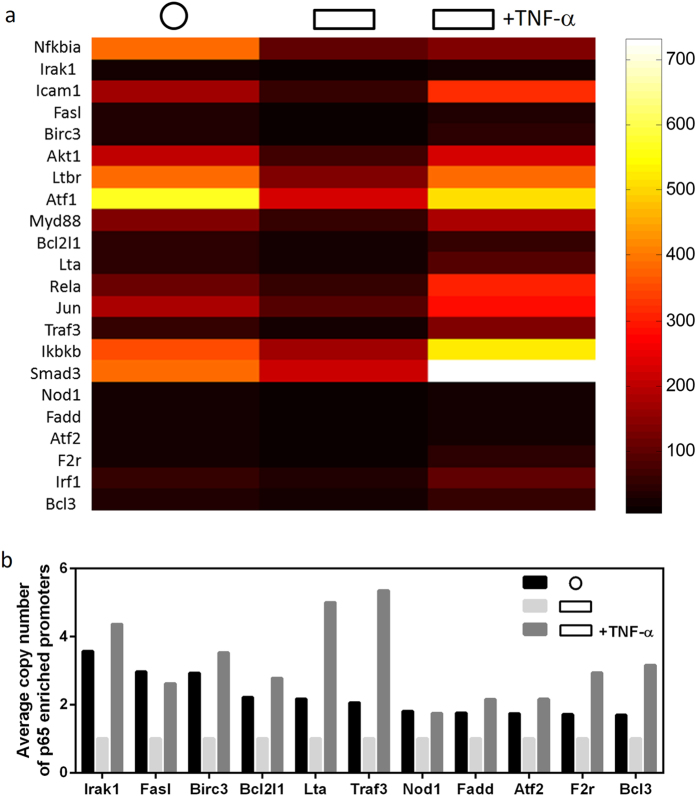
EpiTect ChIP qRCR analysis reveals particular genes with differential enrichment of p65 at the promoters in response to geometric confinement or cytokine induction. (**a**) Color map of genes with differential p65 enrichment. The cutoff of fold change is 1.5. (**b**) Bar graph quantifying the p65 enrichment of genes indicated with darker colors in (**a**).

Under different conditions, the overall trend in the p65 promoter occupancy was similar to the trend of the chromosomal contacts level targeted by p65 (Supplementary Fig. 10, Fig. 4). Among the ~80 known p65 target genes, we found that 22 differentially associated with p65 in response to geometric confinement and cytokine induction. Changes in association were defined by a fold change cutoff of 1.5 (Fig. 5). More interestingly, the genes that were sensitive to geometry confinement and cytokine induction generally were more enriched with p65 compared to those with less sensitivity (Supplementary Fig. 10). This suggests that the promoters of these genes are more likely to be contained in the chromosomal contacts targeted by p65. These results suggest that the chromosomal contacts targeted by p65, and visualized in less-spread cells or cells with TNF-α treatment, contain promoter sites recognized by p65. Similarly, using libraries of primers for other groups of genes, one could know the genetic information of chromosomal contacts targeted by various transcription factors.

## Discussion

In this manuscript, we have described a method to systematically visualize functional chromosomal contacts by combining modified open chromatin spreads and superresolution microscopy. This method has important applications for directly assessing the nanoscale contacts that are formed between genes within the 3D nuclear architecture. A number of studies have visualized the co-clustering of genes using DNA and RNA FISH^13^,^14^,^29^,^30^. Such functional clustering is believed to be important in gene co-regulation, since the binding of the specific transcription factors to their respective genes, together with the RNA pol2 machinery, is more likely to occur when all factors are in spatial proximity. However, as such spatial clustering could not be visualized using existing technology, these associations could, until now, only be indirectly correlated to co-regulation and co-clustering.

Our method takes advantage of the fact that chromosomal contacts are tightly fixed by active transcription machinery and transcription factors. After dilution, non-specific contacts, which were present due to the crowded nuclear environment, were removed. Functional chromosomal contacts were spread onto glass slides. DNA was stained, and antibodies to active transcription machinery and transcription factors were used to visualize these structures. We employed a variant of PALM imaging known as BALM, which enables visualization of intact chromatin fibers at the resolution of ~30 nm^20^. With this method, we revealed the nanoscale structures of serum responsive clusters, YAP, and NF-κB gene clusters and quantified the level of these structures depending on the functional state of the cells.

To further validate if the chromosomal contacts contain specific promoter sites, we purified the contacts using antibody-coated magnetic beads, and carried out EpiTect ChIP assays using magnetic beads coated with antibodies to p65. These revealed that the clusters were enriched for p65 transcription factors. Furthermore, the level of p65 bound clusters correlated with promoter occupancy of p65 target genes.

Taken together, we describe a method to visualize functional gene clusters within the cell nucleus, and show that these clusters are correlated with the promoter occupancy of specific transcription factors. Our method opens a novel platform to directly visualize transcription-dependent nanoscale clustering of genes. We suggest that such imaging methods, combined with the sequencing of gene clusters, will provide avenues to map the spatial configuration of genes that are in physical proximity in cell type specific transcription regulatory networks. Such methods will be valuable in analyzing gene clusters in normal cells and in cells related to specific disease states.

## Methods

### Cell culture, micropatterning, and cytokine induction

NIH 3T3 fibroblast cells were cultured in low glucose Dulbecco’s modified Eagle’s medium (DMEM, GIBCO, New York, USA) supplemented with 10% fetal bovine serum (FBS, Gibco) and 1% (vol/vol) penicillin streptavidin (GIBCO, New York, USA) at 37 °C in 5% CO_2_. Glass slides were cleaned with 100% ethanol, and spin-coated with Polydimethylsiloxane (PDMS). This was followed by microprinting with fibronectin-coated PDMS stamps. After that, 65,000 cells were seeded for 10–15 min on fibronectin islands with different shapes. Non-adhered cells were removed and the remaining cells were washed once with DMEM, and incubated for 3 hrs at 37 °C in 5% CO2. In cytokine induction experiments, 25 ng/ml TNF-a (Sigma-Aldrich, USA) was added to cells after 3hrs incubation for 30 min.

### Serum starvation and stimulation

NIH3T3 cells were starved by culturing them in low glucose Dulbecco’s modified Eagle’s medium (DMEM, GIBCO, New York, USA) supplemented with 1% fetal bovine serum (FBS, Gibco) and 1% (vol/vol) penicillin streptavidin (GIBCO, New York, USA) at 37 °C in 5% CO_2_ for 36hrs. Serum stimulation was achieved by replacing the serum-poor DMEM with normal DMEM supplemented with 10% FBS for 12 hrs.

### Preparation for digested chromatin spreads

After NIH3T3 cells were treated with serum starvation/ stimulation, geometry confinement, or cytokine induction, the cytoplasm was removed by incubating cells with lysis buffer containing 2 mM MgCl_2_, 10 mM Tris-HCl (pH 7.4), 1X protease inhibitor cocktail, and 1% Triton for 2 min on ice. Isolated nuclei were gently washed with digestion buffer twice, and then incubated with FastDigest HindIII (Thermo Fisher Scientific, USA) containing 1X protease inhibitor cocktail for 20 min at 37 °C. After that, the nuclei were incubated with 1% BSA, containing 1X protease inhibitor cocktail for 1hr at room temperature. This was followed by incubation with primary antibodies for RNA Polymerase II CTD repeat YSPTSPS (phosphor S5) (Abcam- ab5131, 1:500), RNA Polymerase II CTD repeat YSPTSPS (phosphor S5) (Millipore- 04–1572, 1:500), Serum Response Factor, SRF (Santa Cruz biotechnology, USA, sc-335, 1:100), NF-kB p65 (Cell Signaling Technology, 8284, 1:300), and YAP1 (Abcam ab56701, 1:200), and corresponding secondary antibodies. Labeled nuclei were then swollen with deionized (DI) water for 10 min, and rinsed twice with blinking buffer (50 mM Tris-HCl, 50 nM NaCl, 1 mM EDTA, 1 mM Methyl Viologen (Aldrich, USA), 10 mM L-Ascorbic acid (Sigma, USA), pH 7.5) together with 100 ng/ml of YOYO-1 (Invitrogen, USA). Following nuclei rupture, either direct confocal imaging, or superresolution imaging was carried out. All experiments were performed in triplicate.

### Confocal imaging and image analysis

Images were captured with a Nikon A1R microscope using a 100x, 1.4 NA oil objective. Imaging conditions were kept the same in all of the experiments.

### Super-resolution imaging and image analysis

Super-resolution imaging was performed on a Zeiss Elyra P.1 microscope, equipped with an oil-immersion objective (alpha “Plan-Apochromat” 100X/1.46 Oil DIC) and total internal fluorescence (TIRF) illumination. Emitted fluorescence was collected by the same objective and captured by an Andor iXon 897 back-thinned EMCCD camera. Integration time per frame was 33 ms at full laser power for the 488 channel, and 50 ms for the 561 and 647 channels. The laser power of 561 nm and 647 nm required adjustment according to the staining condition. Typically 10,000 frames were collected, which corresponded to a measurement duration of 5–10 min for each channel. XY drift and channel misalignment was corrected by localizing 0.2-μm TetraSpeck^TM^ beads (Invitrogen, USA) immobilized on the sample coverslip. For super-resolution data analysis, the raw data was processed using Zeiss Zen software to detect single-molecule events above the background noise (more details are described in^18^). After reconstruction, a super-resolution image and a table containing the x-y coordinates of all the single-molecule events were obtained. In the post-processing step, events which were above the 20 nm localization limit were discarded. A super-resolution (SR) image was generated by fitting each event with the Gaussian function. The exported SR images were then processed in ImageJ.

### Chromosome contact pull down and EpiTect ChIP qPCR

#### Fixing and preparation for immunostaining

NIH3T3 cells (approximately one million) that were geometrically confined, and treated with cytokines, were fixed with 2% formaldehyde for 5 min at room temperature (RT) followed by quenching with 127 mM glycine for 10 min at RT. Cells were washed with Phosphate-buffered Saline (PBS). The nuclei were prepared in lysis buffer (10 mM Tris-HCl (pH 8), 10 mM NaCl, 0.2% IGEPAL CA-630(Sigma)) with protease inhibitor cocktail (Roche) for 30 min on ice with intermittent agitation. Nuclei were washed with 1x Fast Digest (FD) buffer (Thermo Fisher Scientific). 400 μl of 1 x FD buffer and 6 μl of 20% SDS was added to the nuclei and incubated at 37 °C for 60 min with constant agitation. 40 μl of 20% Triton X-100 was added and incubated at 37 °C for 60 min with constant agitation. 30 μl of HindIII (50 U/μl; Thermo Fisher Scientific) was added and incubated at 37 °C for overnight with constant agitation. Nuclei were washed with PBS and blocked with 5% BSA for 1 hr at RT before being immunostained. Nuclei were washed with 5% BSA, scraped and collected in a tube.

#### Coupling with beads

Dynabeads coupled with Anti-Rabbit secondary antibody (M-280; Thermo Fisher Scientific) were resuspended in 1 ml of Washing Buffer (Ca2 + and Mg2 + free (PBS), supplemented with 0.1% bovine serum albumin (BSA) and 2 mM EDTA, pH 7.4). With the help of a DynaMag^TM^−2 Magnet, the Dynabeads were washed. 5% BSA and NF-κB p65 Rabbit mAb (Cell Signalling Technology) were added to the beads and incubated with gentle tilting and rotation at RT for one hour. The unbound NF-κB p65 Rabbit mAb was removed using a DynaMag^TM^-2 Magnet. Dynabeads were washed with 5% BSA to ensure all unbound NF-kB p65 Rabbit mAb was removed.

These Dynabeads were then resuspended in the nuclei in 5% BSA and incubated for over 12 hours at 4 °C. The product obtained after the incubation was a tertiary complex comprised of Dynabeads coated with Anti-Rabbit secondary antibody, bound to NF-κB p65 Rabbit mAb, which was further bound to chromatin associated with NF-κB p65. The beads were washed with PBS, to ensure that the chromatin that was not associated with NF-κB p65 was washed off.

#### Reverse crosslinking

Reverse crosslinking was performed by incubating the pulled-down contacts with 5 μl of Proteinase K (PK; Thermo Fisher Scientific) in 200 μl of PK buffer (30 mM Tris (pH8.0), 10 mM EDTA, 1% SDS) incubated at 65 °C for at least 90 min. Using DynaMag^TM^-2 Magnet the supernatant was separated from the Dynabeads. 50 μL of PK buffer was added to the bead fraction to elute any remaining DNA. The supernatant collected was purified using Qiagen PCR clean up to concentrate the DNA. This DNA was further amplified using REPLI-g Single Cell Kit (Qiagen). The amplified DNA was analysed using EpiTect ChIP qPCR array (Qiagen).

### Statistical analysis

Statistical significance was tested using an independent two-tailed student t test in Prism 6.0 (GraphPad). We repeated experiments a minimum of three times with sufficient n numbers for each repeat to be confident that reported results are representative. Error bars on graphs show ± standard error of the means (s.e.m.).

## Additional Information

**How to cite this article**: Yejun, W. *et al*. Superresolution imaging of nanoscale chromosome contacts. *Sci. Rep.* **7**, 42422; doi: 10.1038/srep42422 (2017).

**Publisher's note:** Springer Nature remains neutral with regard to jurisdictional claims in published maps and institutional affiliations.

## Supplementary Material

Supplementary Materials

## References

[b1] Cremer T. & Cremer C. Chromosome territories, nuclear architecture and gene regulation in mammalian cells. Nature reviews genetics 2, 292–301 (2001). 10.1038/35066075 11283701

[b2] Misteli T. Beyond the sequence: cellular organization of genome function. Cell 128, 787–800 (2007). 17320514 10.1016/j.cell.2007.01.028

[b3] Bickmore W. A. & van Steensel B. Genome architecture: domain organization of interphase chromosomes. Cell 152, 1270–1284 (2013). 23498936 10.1016/j.cell.2013.02.001

[b4] Bartman C. R. & Blobel G. A. In Cold Spring Harbor symposia on quantitative biology. 027359 (Cold Spring Harbor Laboratory Press).

[b5] Pombo A. & Dillon N. Three-dimensional genome architecture: players and mechanisms. Nature reviews Molecular cell biology 16, 245–257 (2015). 25757416 10.1038/nrm3965

[b6] Cremer M. . Non-random radial higher-order chromatin arrangements in nuclei of diploid human cells. Chromosome research 9, 541–567 (2001). 11721953 10.1023/a:1012495201697

[b7] Bolzer A. . Three-dimensional maps of all chromosomes in human male fibroblast nuclei and prometaphase rosettes. PLoS Biol 3, e157 (2005). 15839726 10.1371/journal.pbio.0030157 PMC1084335

[b8] Iyer K. V. . Modeling and experimental methods to probe the link between global transcription and spatial organization of chromosomes. PloS one 7, e46628 (2012). 23049710 10.1371/journal.pone.0046628 PMC3462193

[b9] Maharana S. . Chromosome intermingling‚Äîthe physical basis of chromosome organization in differentiated cells. *Nucleic acids research*, gkw131 (2016). 10.1093/nar/gkw131 PMC5603959 26939888

[b10] Maharana S. . Chromosome intermingling—the physical basis of chromosome organization in differentiated cells. *Nucleic acids research*, gkw131 (2016). 10.1093/nar/gkw131 PMC5603959 26939888

[b11] Kaufmann S. . Inter-chromosomal contact networks provide insights into Mammalian chromatin organization. PloS one 10, e0126125 (2015). 25961318 10.1371/journal.pone.0126125 PMC4427453

[b12] Khrameeva E. E., Mironov A. A., Fedonin G. G., Khaitovich P. & Gelfand M. S. Spatial proximity and similarity of the epigenetic state of genome domains. PloS one 7, e33947 (2012). 22496774 10.1371/journal.pone.0033947 PMC3319547

[b13] Schoenfelder S. . Preferential associations between co-regulated genes reveal a transcriptional interactome in erythroid cells. Nature genetics 42, 53–61 (2010). 20010836 10.1038/ng.496 PMC3237402

[b14] Osborne C. S. . Active genes dynamically colocalize to shared sites of ongoing transcription. Nature genetics 36, 1065–1071 (2004). 15361872 10.1038/ng1423

[b15] Noordermeer D. . The dynamic architecture of Hox gene clusters. Science 334, 222–225 (2011). 21998387 10.1126/science.1207194

[b16] Fanucchi S., Shibayama Y., Burd S., Weinberg M. S. & Mhlanga M. M. Chromosomal contact permits transcription between coregulated genes. Cell 155, 606–620 (2013). 24243018 10.1016/j.cell.2013.09.051

[b17] Matsuda A. . Condensed mitotic chromosome structure at nanometer resolution using PALM and EGFP-histones. PloS one 5, e12768 (2010). 20856676 10.1371/journal.pone.0012768 PMC2939896

[b18] Zessin P. J., Finan K. & Heilemann M. Super-resolution fluorescence imaging of chromosomal DNA. Journal of Structural Biology 177, 344–348 (2012). 22226957 10.1016/j.jsb.2011.12.015

[b19] Boettiger A. N. . Super-resolution imaging reveals distinct chromatin folding for different epigenetic states. Nature 529, 418–422 (2016). 26760202 10.1038/nature16496 PMC4905822

[b20] Schoen I., Ries J., Klotzsch E., Ewers H. & Vogel V. Binding-activated localization microscopy of DNA structures. Nano letters 11, 4008–4011 (2011). 21838238 10.1021/nl2025954

[b21] Doksani Y., Wu J. Y., de Lange T. & Zhuang X. Super-resolution fluorescence imaging of telomeres reveals TRF2-dependent T-loop formation. Cell 155, 345–356 (2013). 24120135 10.1016/j.cell.2013.09.048 PMC4062873

[b22] Wang Y., Maharana S., Wang M. D. & Shivashankar G. Super-resolution microscopy reveals decondensed chromatin structure at transcription sites. Scientific reports 4 (2014). 10.1038/srep04477 PMC3966049 24667378

[b23] Dekker J. The three’C’s of chromosome conformation capture: controls, controls, controls. Nature methods 3, 17–21 (2006). 16369547 10.1038/nmeth823

[b24] Betzig E. . Imaging intracellular fluorescent proteins at nanometer resolution. Science 313, 1642–1645 (2006). 16902090 10.1126/science.1127344

[b25] Huang B., Babcock H. & Zhuang X. Breaking the diffraction barrier: super-resolution imaging of cells. Cell 143, 1047–1058 (2010). 21168201 10.1016/j.cell.2010.12.002 PMC3272504

[b26] Maeshima K., Hihara S. & Eltsov M. Chromatin structure: does the 30-nm fibre exist *in vivo*? Current opinion in cell biology 22, 291–297 (2010). 20346642 10.1016/j.ceb.2010.03.001

[b27] Dupont S. . Role of YAP/TAZ in mechanotransduction. Nature 474, 179–183 (2011). 21654799 10.1038/nature10137

[b28] Jain N., Iyer K. V., Kumar A. & Shivashankar G. Cell geometric constraints induce modular gene-expression patterns via redistribution of HDAC3 regulated by actomyosin contractility. Proceedings of the National Academy of Sciences 110, 11349–11354 (2013). 10.1073/pnas.1300801110 PMC3710882 23798429

[b29] Osborne C. S. . Myc dynamically and preferentially relocates to a transcription factory occupied by Igh. PLoS Biol 5, e192 (2007). 17622196 10.1371/journal.pbio.0050192 PMC1945077

[b30] Xu M. & Cook P. R. Similar active genes cluster in specialized transcription factories. The Journal of cell biology 181, 615–623 (2008). 18490511 10.1083/jcb.200710053 PMC2386102

