# Supplementary material for: Superresolution imaging of nanoscale chromosome contacts
Source: Sci Rep. 2017 Feb 10;7:42422. doi: 10.1038/srep42422 (PMC5301241; doi:10.1038/srep42422)
Supplement: Supplementary Materials [file srep42422-s1.pdf]

Superresolution imaging of nanoscale chromosome contacts

Authors: Yejun Wang[1], Prasuna Ratna [1, 2], G.V. Shivashankar[1, 2*]

Affiliations:

[1]Mechanobiology Institute and Department of Biological Sciences,

National University of Singapore 117411, Singapore.

[2]FIRC Institute for Molecular Oncology (IFOM), Milan 20139, Italy.

*Correspondence to: shiva.gvs@gmail.com

Supplementary figure legends

Supplementary Figure 1. Conventional microscopy imaging of digested chromatin fragments. (a) TIRF images of chromatin spreads with and without digestion. Scale bar: 10 μm. (b) Length distribution of digested chromatin fragments. Inset: zoomed in image of the region outlined by the orange box in (a). Scale bar: 1 μm.

Supplementary Figure 2. Digested chromatin spreads prepared with/ without fixation. Confocal images of intact nuclei and TIRF images of digested chromatin spreads with or without fixation. Scale bar: 10 μm.

Supplementary Figure 3. Superresolution microscopy imaging of digested chromatin fragments reveals chromosomal contacts. (a) TIRF image of digested chromatin fragments. Scale bar: 10 μm. (b) Zoomed in TIRF image of the region outlined by a white box in (a). Inset: zoomed in images of the regions outlined by white boxes in (b). (c) Zoomed in superresolution image of the region outlined by a white box in (a). Scale bar: 2 μm. Inset: zoomed in images of the regions outlined by white boxes in (c). Scale bar: 200 nm. (d) Width distribution of chromosomal contacts visualized by superresolution microscopy.

Supplementary Figure 4. Three-color superresolution imaging of chromosomal contacts with 5S RNA pol2 and the transcription factor SRF in serum +/- system. (a) Three-color superresolution image of chromosomal contacts (green), 5S RNA pol2 (red), and SRF (blue). Scale bar: 500 nm. Insets: zoomed in images of the regions outlined by white boxes. Scale bar: 200 nm. (b)

Bar graph quantifying the percentage of chromosomal contacts associated with both 5S RNA pol2 and SRF. Data is given as mean ± SD with $10<n<20$. $^{**}P<0.01$; Two sample student's t test.

Supplementary Figure 5. Nuclear shape was maintained after cytoplasm removal. Bright field images of cells and nuclei on either anisotropic (rectangle) or isotropic (circle) substrates.

Supplementary Figure 6. Conventional microscopy images of digested chromatin fragments with 5S RNA pol2 and the transcription factor YAP under geometric confinement. (a) Three-color TIRF images of chromatin fragments (green), 5S RNA pol2 (red), and YAP (blue). Scale bar: 5 μm. (b) Bar graph quantifying the ratio of YAP and 5S RNA pol2 total intensity. Data is given as mean ± SD with $10<n<20$. $^{***}P<0.001$; Two sample student's t test. The rectangle refers to big anisotropic substrate, and the circle refers to small isotropic substrate.

Supplementary Figure 7. Conventional microscopy images of digested chromatin fragments with 5S RNA pol2 and the transcription factor SRF under geometric confinement. (a) Three-color TIRF images of chromatin fragments (green), 5S RNA pol2 (red), and SRF (blue). Scale bar: 10 μm. (b) Bar graph quantifying the ratio of SRF and 5S RNA pol2 total intensity. Data is given as mean ± SD with $10<n<20$. $^{***}P<0.001$; Two sample student's t test. The rectangle refers to big anisotropic substrate, and the circle refers to small isotropic substrate.

Supplementary Figure 8. Nuclear localization of p65 regulated by cell geometry and cytokine treatment. (a) Representative images of the nucleus, and p65. Scale bar: 5 μm. (b) Bar graph quantifying the nuclear to cytoplasmic ratio (N2C) of p65 in three conditions normalized to the condition in anisotropic substrates. Data is presented as mean ± SE with 20 < n < 30. *** $P<0.001$; Two sample student's t test. The rectangle refers to big anisotropic substrate, and the circle refers to small isotropic substrate.

Supplementary Figure 9. Conventional microscopy images of digested chromatin fragments with 5S RNA pol2 and the transcription factor p65 under geometric confinement and cytokine induction. (a) Three-color TIRF images of chromatin fragments (green), 5S RNA pol2 (red), and p65 (blue). Scale bar: 10 μm. (b) Bar graph quantifying the ratio of p65 and 5S RNA pol2 total intensity. Data is given as mean ± SD with $10<n<20$. *$P<0.05$; one-way ANOVA test. The rectangle refers to big anisotropic substrate, and the circle refers to small isotropic substrate.

Supplementary Figure 10. Color map of the whole set of genes with differential p65 enrichment. The rectangle refers to big anisotropic substrate, and the circle refers to small isotropic substrate.

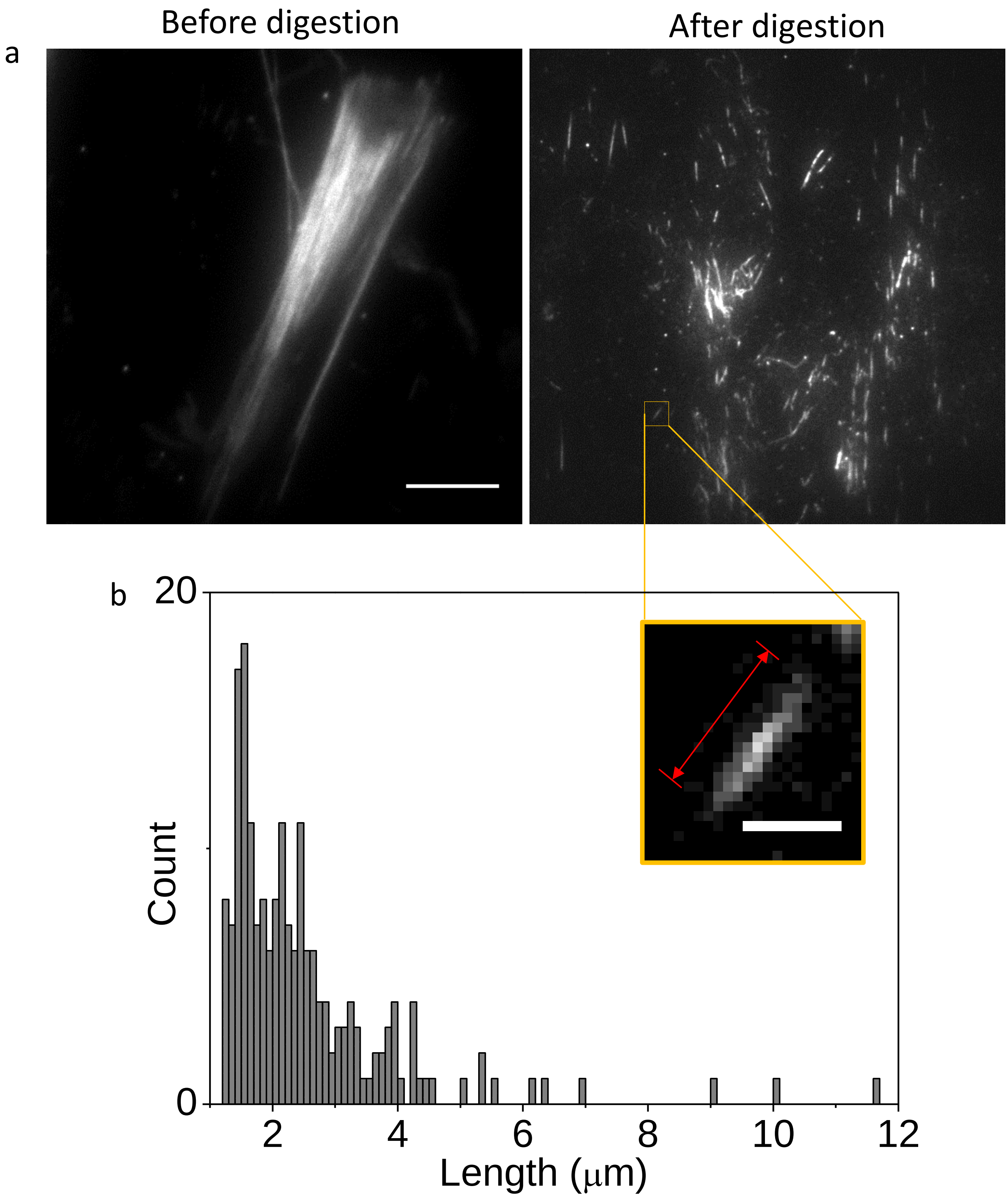
Before digestion
After digestion
a
b
20
0
Count
2
4
6
8
10
12
Length (μm)

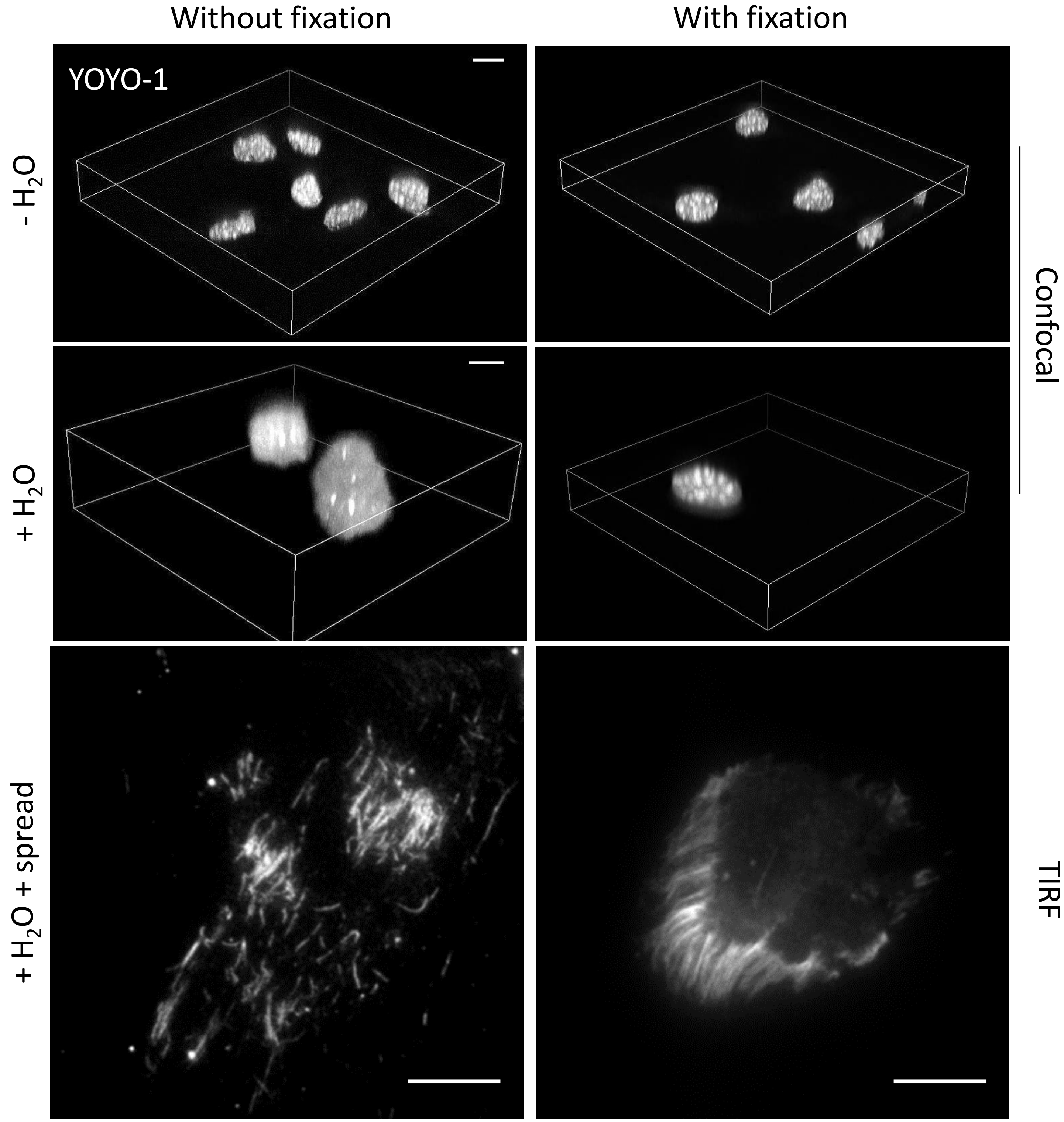

Without fixation
With fixation
YOYO-1
- H_2O
+ H_2O
+ H_2O + spread
Confocal
TIRF

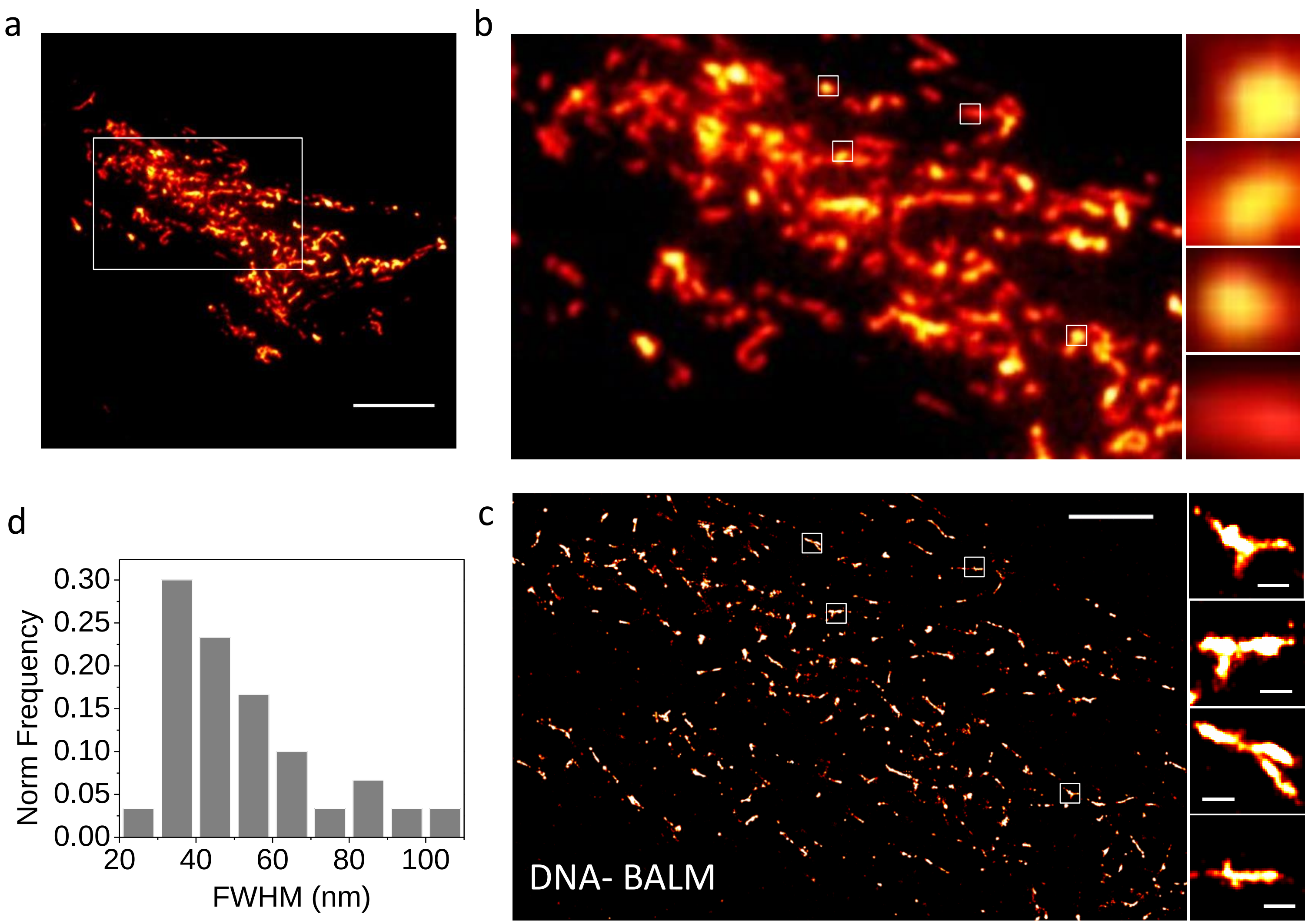

a
b
c
d
0.30
0.25
0.20
0.15
0.10
0.05
0.00
Norm Frequency
20
40
60
80
100
FWHM (nm)
DNA- BALM

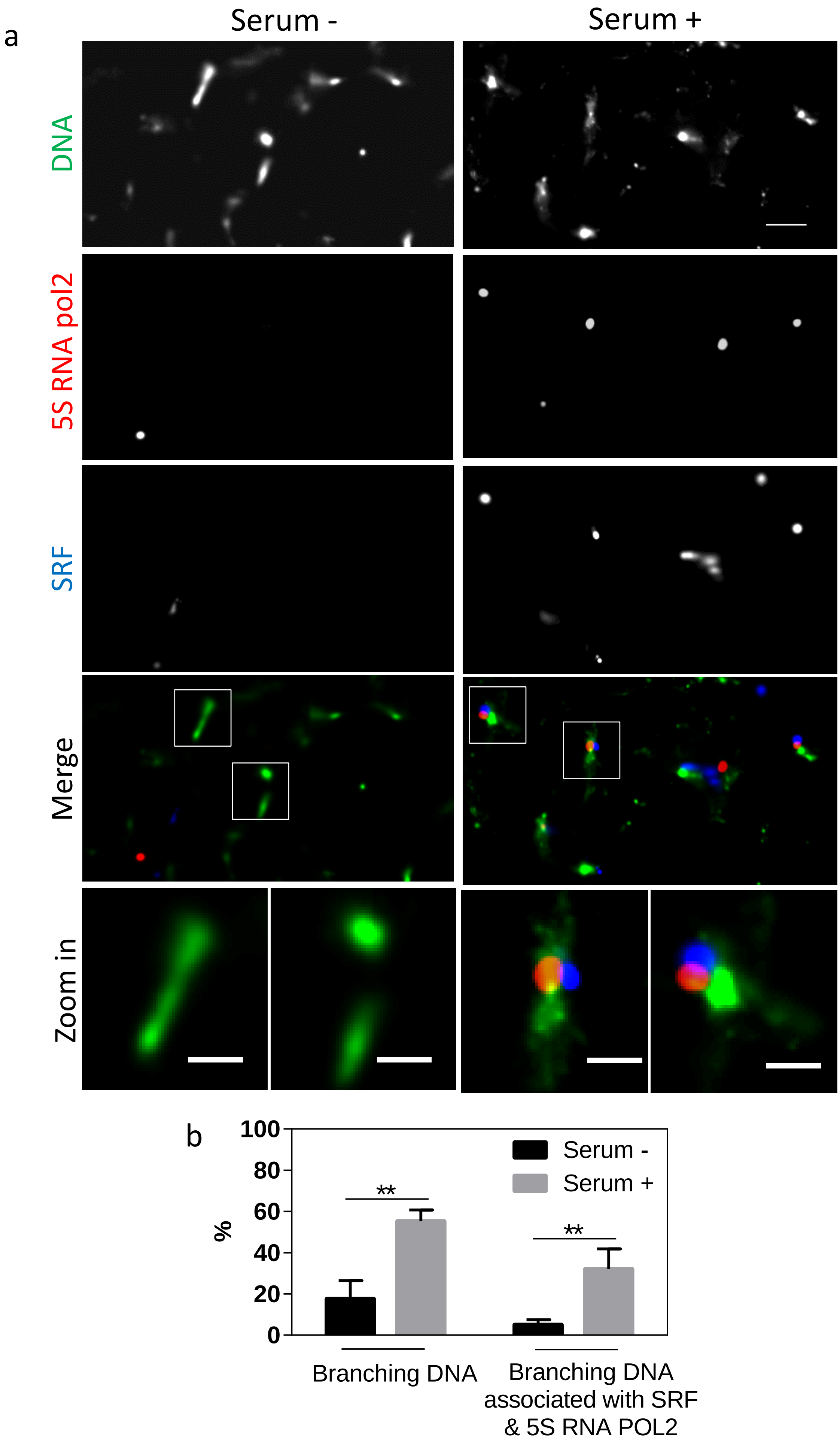
a
Serum -
Serum +
DNA
5S RNA pol2
SRF
Merge
Zoom in
b
100
80
60
40
20
0
%
Serum -
Serum +
**
**
Branching DNA
Branching DNA associated with SRF & 5S RNA POL2

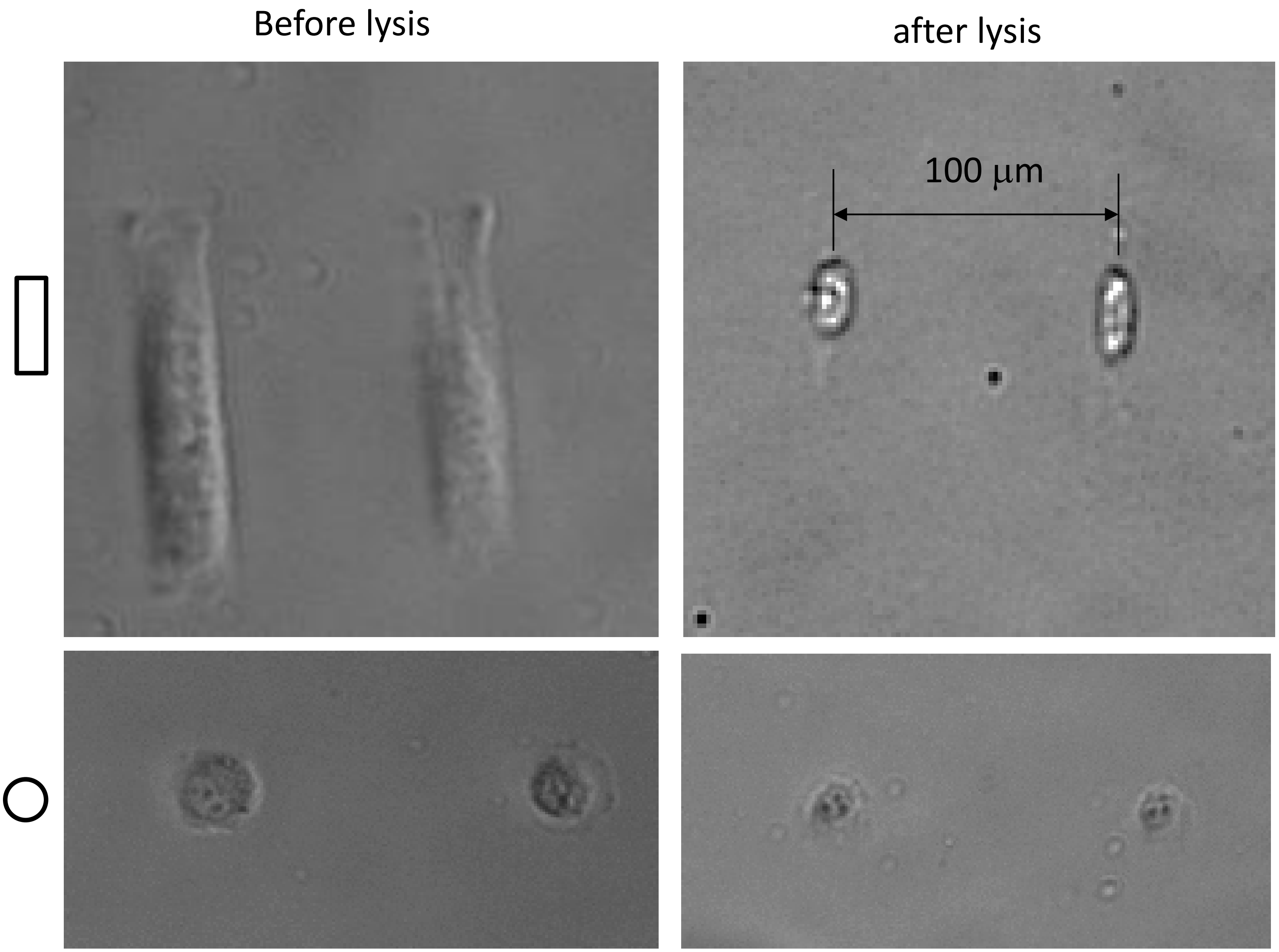
Before lysis
after lysis
100 μm

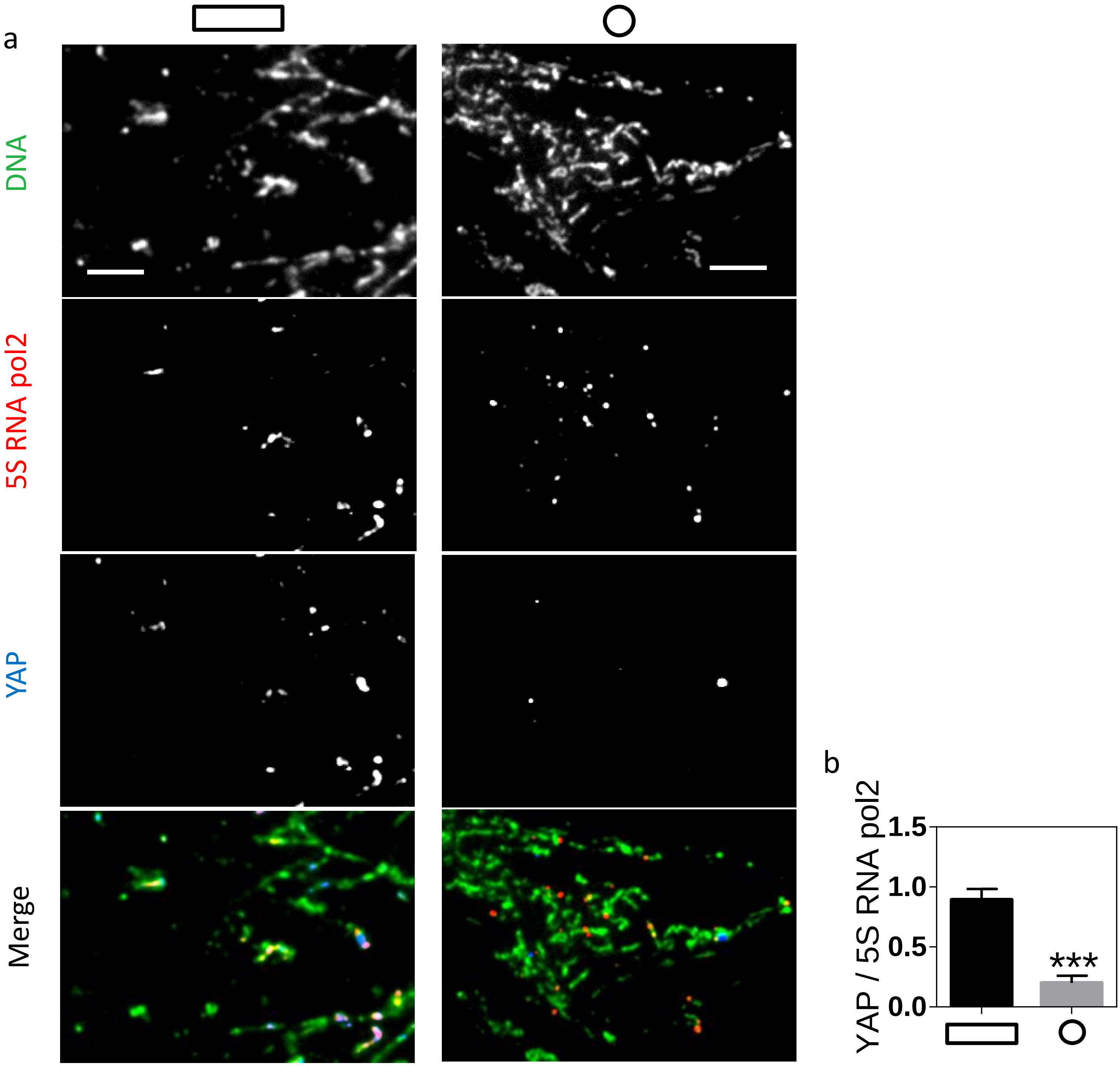

a
DNA
5S RNA pol2
YAP
Merge
b
YAP / 5S RNA pol2
1.5
1.0
0.5
0.0
***

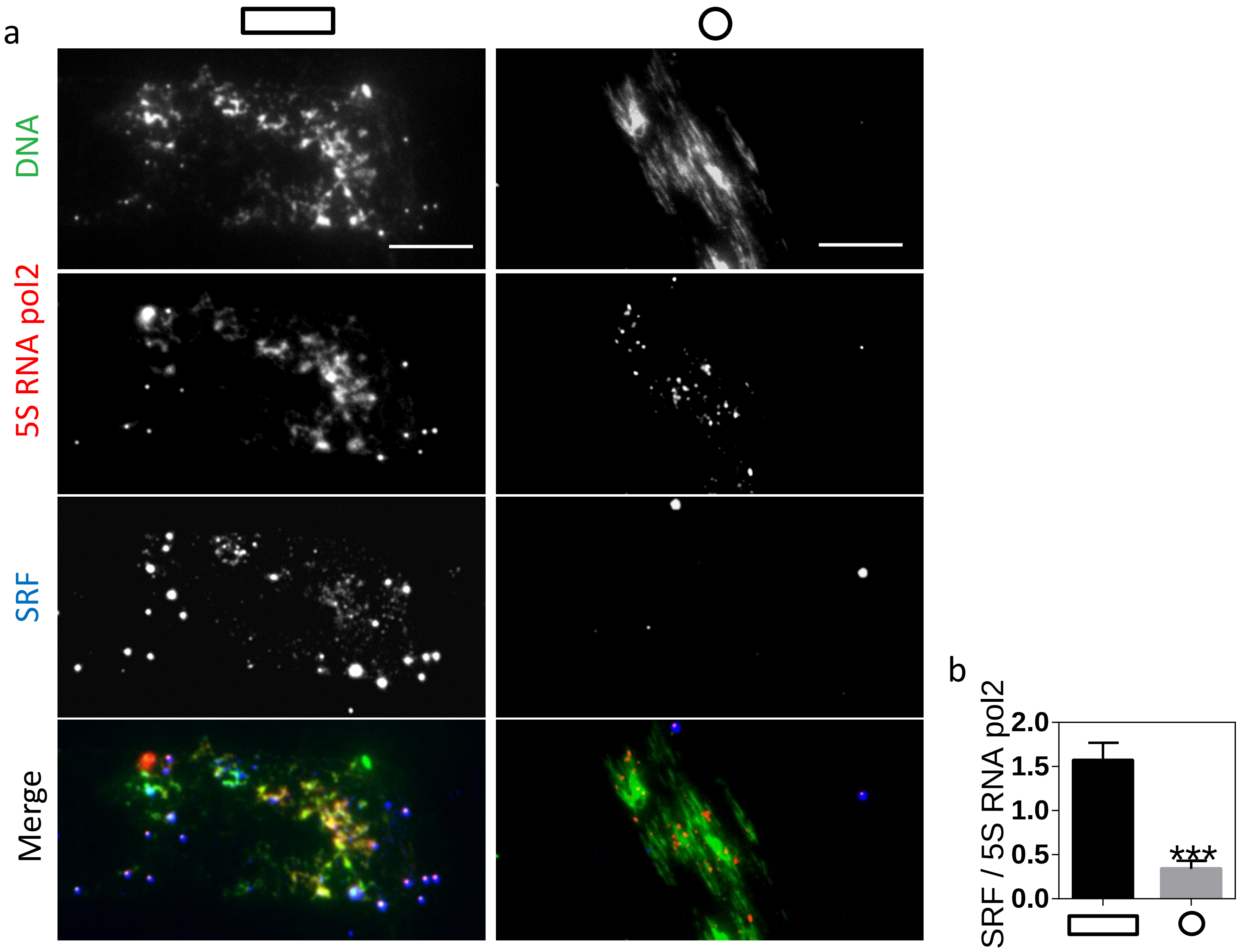

a
DNA
5S RNA pol2
SRF
Merge
b
SRF / 5S RNA pol2
2.0
1.5
1.0
0.5
0.0
***

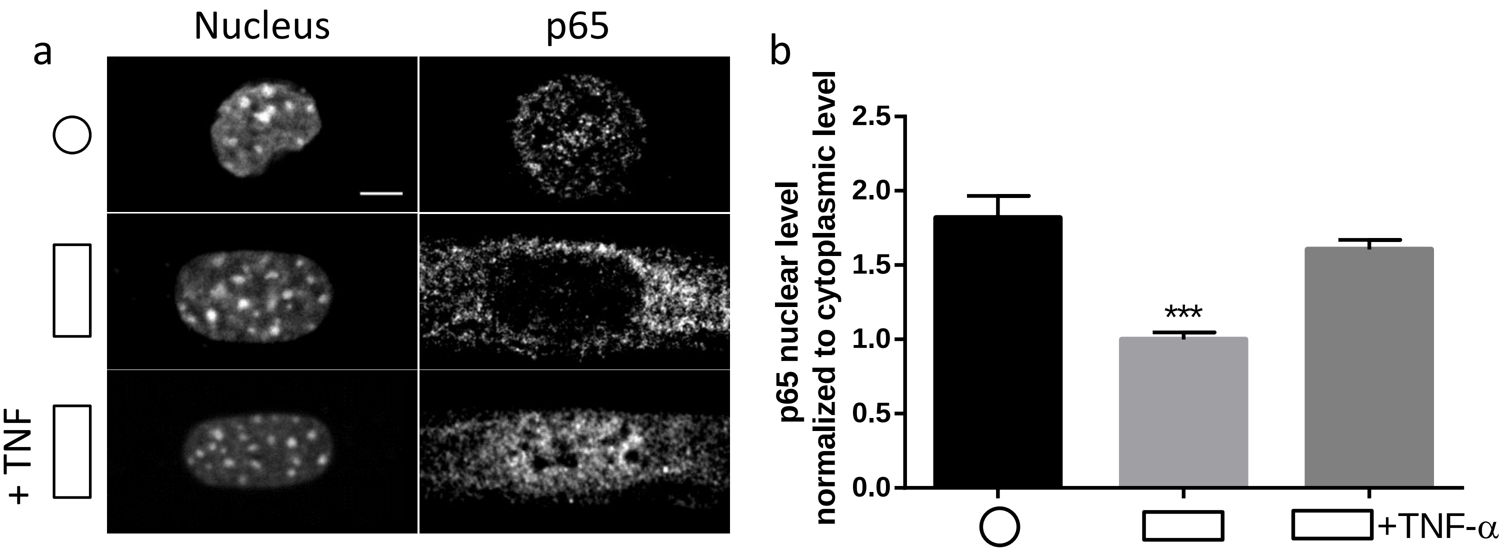

a
Nucleus
p65
+ TNF
b
p65 nuclear level normalized to cytoplasmic level
2.5
2.0
1.5
1.0
0.5
0.0
***
+TNF-α

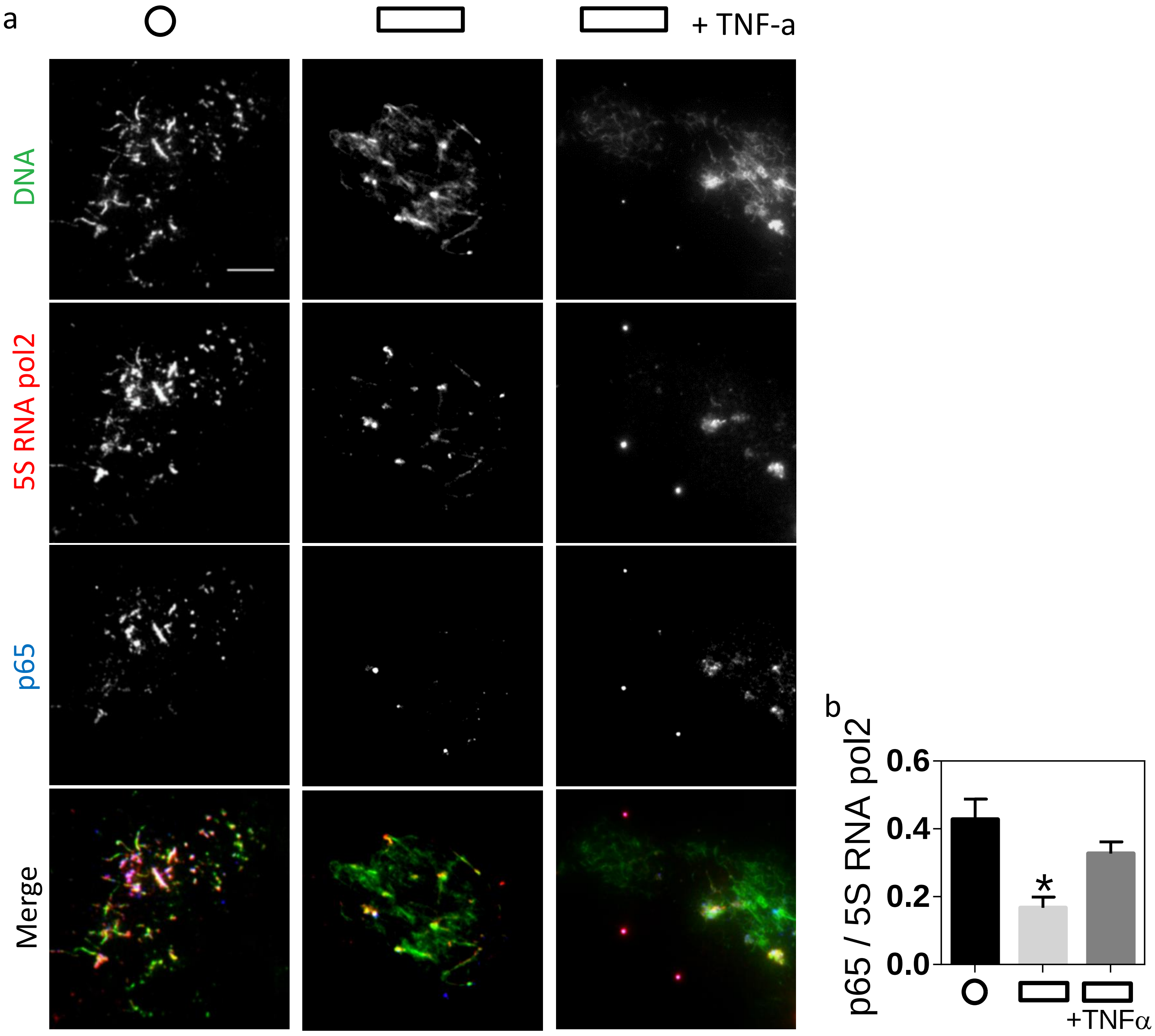

a
+ TNF-a
DNA
5S RNA pol2
p65
Merge
b
0.6
0.4
0.2
0.0
p65 / 5S RNA pol2
*
+TNFα

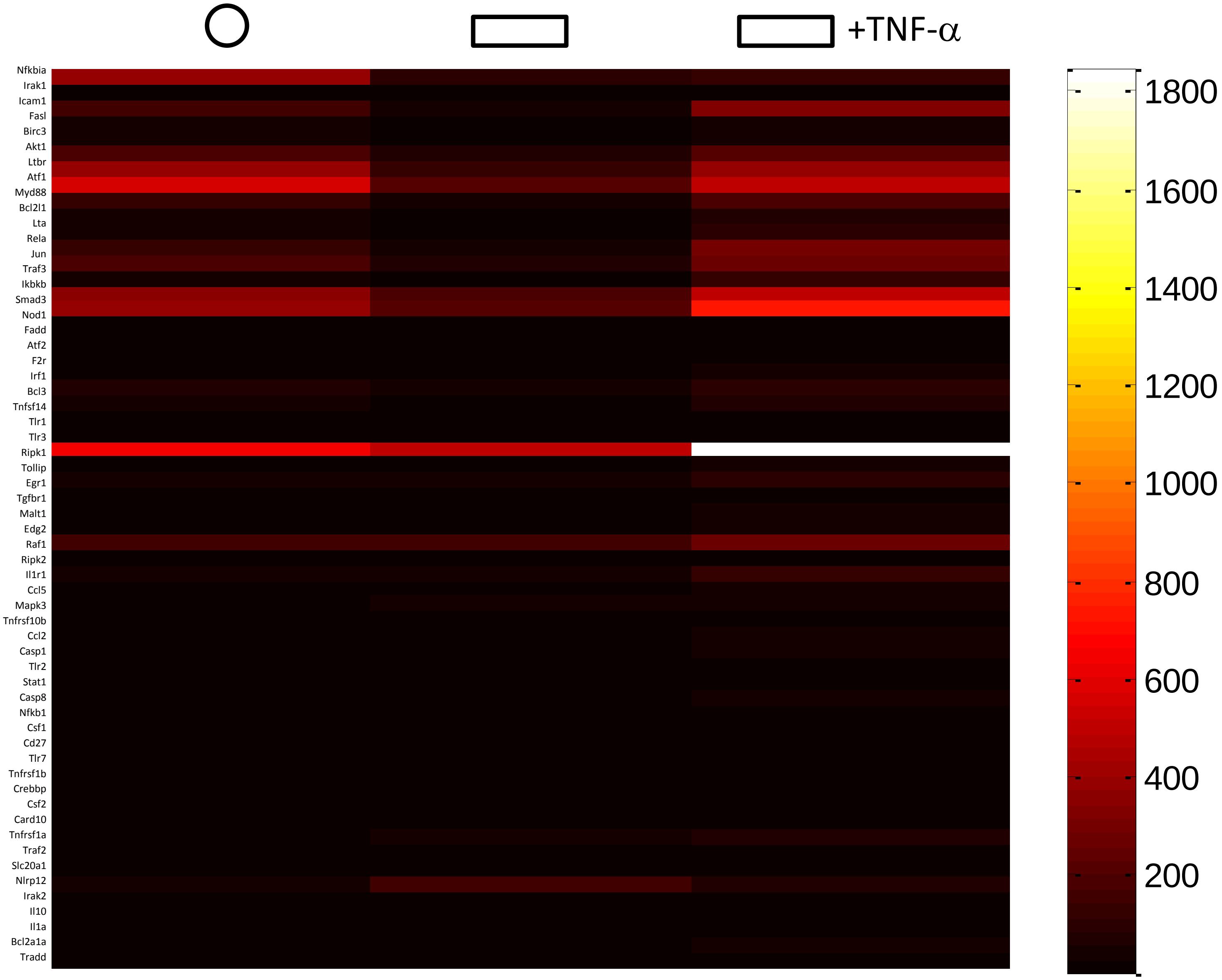
+TNF-α
Nfkbia
Irak1
Icam1
Fasl
Birc3
Akt1
Ltbr
Atf1
Myd88
Bcl2l1
Lta
Rela
Jun
Traf3
Ikbkb
Smad3
Nod1
Fadd
Atf2
F2r
Irf1
Bcl3
Tnfsf14
Tlr1
Tlr3
Ripk1
Tollip
Egr1
Tgfbr1
Malt1
Edg2
Raf1
Ripk2
Il1r1
Ccl5
Mapk3
Tnfrsf10b
Ccl2
Casp1
Tlr2
Stat1
Casp8
Nfkb1
Csf1
Cd27
Tlr7
Tnfrsf1b
Crebbp
Csf2
Card10
Tnfrsf1a
Traf2
Slc20a1
Nlrp12
Irak2
Il10
Il1a
Bcl2a1a
Tradd
1800
1600
1400
1200
1000
800
600
400
200
